# THE INFLUENCE OF RURALITY AND FRAILTY ON HEALTH OUTCOMES IN OLDER ADULTS

**DOI:** 10.1093/geroni/igad104.2476

**Published:** 2023-12-21

**Authors:** Hillary Spangler, Emma Mitchell, David Lynch, Perry Haaland, John Batsis

**Affiliations:** UNC School of Medicine, Chapel Hill, North Carolina, United States; UNC Chapel Hill, Chapel Hill, North Carolina, United States; UNC School of Medicine, Chapel Hill, North Carolina, United States; UNC Chapel Hill, Chapel Hill, North Carolina, United States; UNC School of Medicine, Chapel Hill, North Carolina, United States

## Abstract

**Background:**

Frailty is a syndrome representing a decline in physical function and an increased vulnerability to stressors. Older adults in rural areas may be at increased risk for frailty and adverse outcomes due to worse overall health. Our aim was to identify how rural/urban residence can influence frailty status.

**Methods:**

We used National Health and Aging Trend Study (2011-2020) data, a cohort of Medicare beneficiaries. Participants were categorized as robust, pre-frail, and frail (Fried’s frailty phenotype). Rural residence included counties outside of a metropolitan statistical area (Office of Management and Budget). Participants were excluded if frailty components or geographical status were incomplete. We used logistic regression analyses for the relationship between adverse outcomes (death, nursing home placement over 2011-2020), rural/urban, and frailty status.

**Results:**

Of 7,393 participants (57.2% female), median age range was 75-80 years and 19.4% were rural residents. Rates of robust, pre-frailty, and frailty were 41.4%, 48.4%, and 10.2%, respectively. There was no difference of frailty status by rural status nor was there a frailty x rural interaction. Older adults with pre-frailty (OR=1.18; 1.05-1.34) and frailty (OR=1.01; 0.82-1.24) had higher risk of residing in rural than urban areas. Rural residence (OR=1.75;1.33-2.24), pre-frailty (OR=1.87;1.54-2.28) and frailty (OR=3.62; 2.79-4.71) had higher risk of adverse outcomes.

**Conclusion:**

Participants with pre-frailty had higher odds of living in a rural area. Similarly, participants in rural areas had higher odds of adverse outcomes. These findings may highlight health care disparities in rural areas and opportunities for system- and individual-level interventions to prevent frailty development.

